# Art-therapy focused on stimulating the emotional and expressive skills of children with special educational needs

**DOI:** 10.1192/j.eurpsy.2022.1409

**Published:** 2022-09-01

**Authors:** L. Lorint

**Affiliations:** Consiliul Județean Cluj Direcția Generală de Asistență Socială și Protecția Copilului, Centrul Comunitar Județean Complex De Servicii Sociale Comunitare Pentru Copii și Adulți Cluj, Cluj-napoca, România, Cluj-Napoca , Romania

**Keywords:** school education, art therapy, school dropout, literacy

## Abstract

**Introduction:**

The link between school education and art therapy is supported by the fact that it comes to supplement the common school education activities of children with special educational requirements with a dual purpose: to complete and fix their specific content; to train and practice as much as possible the students’ minds and critical thinking through artistic means.

**Objectives:**

Prevention of absenteeism and school dropout of the child with special educational requirements through art therapy.

**Methods:**

In art therapy, the most used methods specific to the fields of visual arts are: drawing, painting, icon, modeling on the wheel, but other techniques can be used. Children are the ones who choose their work materials and activities from the offer that the art therapist makes.

**Figure:**
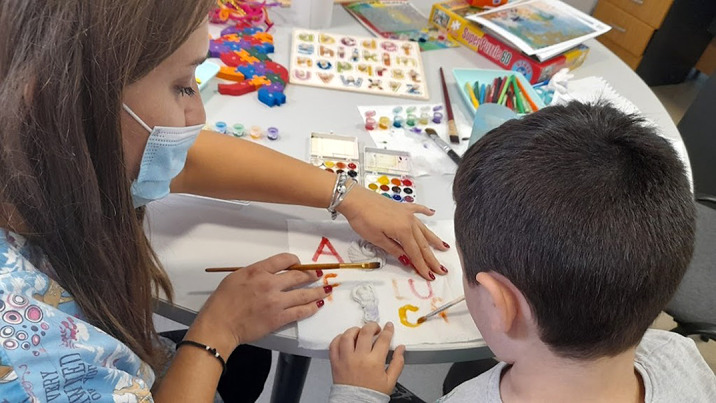

From the offer of materials, the beneficiary prefers to paint porcelain objects and letters. He was challenged to identify letters after which he painted them.

**Results:**

Increasing the feeling of social utility, self-confidence. Improving school situation. Reducing school dropout and literacy.

**Figure:**
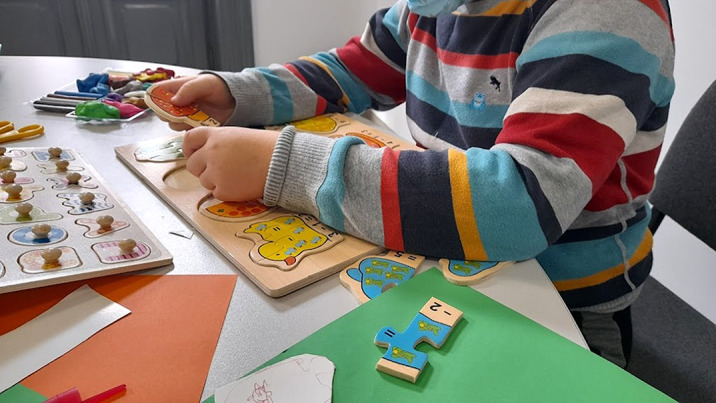

The beneficiary has an interest in letters and numbers and their writing. Form words quickly, easily identifying letters. Create your own games by comparing numbers. Make puzzles with letters and numbers.

**Figure:**
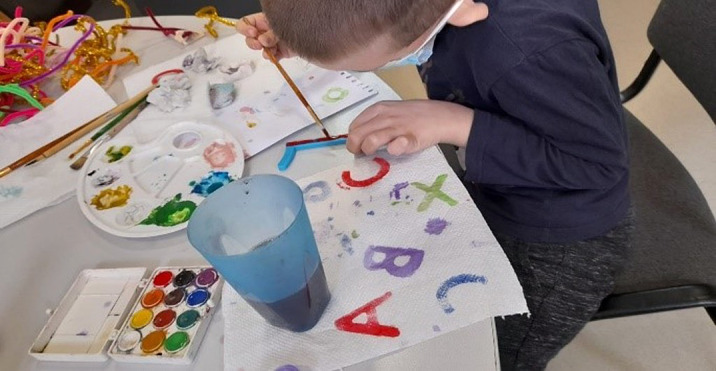

Art education helps the beneficiary to more easily express their emotions, perceptions, desires and way of thinking. Learning numbers and letters becomes a fun activity when using colors.

**Conclusions:**

The child needs education through art, because without it there is an imbalance in the fundamental purpose of man, that of developing harmoniously and multilaterally.

**Disclosure:**

No significant relationships.

